# Tumors of the Lower Jaw. Resection

**Published:** 1858-07

**Authors:** 


### ARTICLE II.

*Tumors of the Lower Jaw. Resection.* With Clinical remarks by PROF. NELATIN. (Translated from the French for the Am. Jour. of Dental Science.)

Two remarkable cases of tumors of the lower jaw were present at the Hôpital des Cliniques, at the commencement of January.

CASE I. Estelle M., seventeen year's old, maker of chasubles, five months since observed a tumor of the size of a small nut, on the horizontal branch of the lower jaw. At this period there had been no particular change in her appearance, no dental or other pain had been felt ; and this absence of the more alarming symptoms had the effect of leaving the patient in complete indifference as to the disease. Of late, the tumor has assumed a greater development ; the cheek of the diseased side pushed out by the protusion of the growing tumor became deformed ; so that the young girl finally decided to put herself under the care of M. Nelatin.

The following observations were made upon her admission. The tumor is about the size of a pigeon's egg ; it is upon both the internal and the external face of the left half of the lower jaw ; it is rounded ; forming, however, a slight elevation below the base of this line. Behind, it nearly reaches the ramus, having for its limit in this direction, the second molar (the third has not yet erupted) and the interstice between the first molar and the second bicuspid in front ; it is a little less in extent upon the inner face of the bone which is altered in character, the mytohyoidean line being deformed. The consistence of this tumor is tolerably uniform ; at first sight, it appeared to be composed of fibrous tissue superimposed upon both faces of the bone. The corresponding teeth are loose, which proves the bony tissue to be diseased.

Desiring to know the exact degree of disorder of the lower jaw in order to determine the plan of operation, Professor Nelatin introduced an acupuncture needle into the tumor. This needle penetrated without difficulty and traversed the bone from side to side in two different directions. The absence of pain and the peculiar form of the tumor, which did not seem to be enclosed in a bony shell, together with the absence of the characteristic crepitation, and its not presenting the usual form of encephaloid, caused the learned practitioner to reject the idea of an affection of that sort. Its slow progress confirmed this negative opinion. We were concerned, therefore, with one of those tumors which profoundly alter the bony tissue, and are somewhat analogous, in their color and density, to muscular fibre. These tumors, originating in the interior of the maxilla, are intermingled with bony spiculæ and lamellæ, and have been designated of late by the name of mycloplaxes.*

* Mycloplaxe (from μυελος, marrow, and πλαξ plates) plates or lamellæ with multiple nuclei of the marrow of bones. M. Robin has given this name to special anatomical element of bone in the normal condition, an element characterized by a very variable form and volume, (0 mm, 020 to 0 mm, 100,) flattened or polyhedric, with borders generally irregular or even dentated, pale and thin, or thick and deep colored, composed of a finely granular mass, studded with ovoid nuclei (from 2 to 3, and as high as 20 or 30.) The nuclei are .009 to .001 mm. long, by mm. .005 to mm. .006 wide, that is, they are about half the size of those of cancer; they may or may not have a nucleolus; when this exists it is smaller than that of the cancerous nuclei, the disposition of the granulations of these elements is not the same as of those of cancer. In the normal condition, the mycloplaxes are found more abundantly in the medulla of the diploe and of the spongy tissue than in that of the long bones. They are proportionally abundant in the medulla of the bony points of new formation in the fœtus. We find them especially adherent to the bony substance of the canal or the areolus filled with medulla, and they model themselves upon the irregularities of this substance. The mycloplaxes which have but one or two nuclei are rare, offering some analogy of form and structure with epithelia, and this element possesses, in the morbid condition, some of the properties of epithelia. In certain cases this element may multiply, and becomes then the principal element of a morbid tissue forming tumors of bones, of the limbs, of the trunk and of the head, which have often been taken for cancer, for want of knowledge of the normal element, which is their point of departure. They may issue from the depths of the bone or from its surface; they seem then to spring from the periosteum, because they have not attacked the bony tissue. In these tumors, the mycloplaxes are sometimes twice as large as in the normal condition, and assumes grotesque forms, while they preserve their structure. The nuclei may or may not increase in size. With the mycloplaxes is found a greater or less quantity of fibro-

Abandoned to themselves, they assume a considerable development, the teeth become loose; important functions are disturbed; for, apart from mastication, which is then performed with difficulty, speech, respiration and deglutition may also be embarrassed. If we add to this that an operation is always necessary, it is evident that it is important, as M. Nelatin said, to decide upon it from the beginning of the affection, the destruction of tissue being less considerable and the consequences less dangerous.

It is very important to know, and this ought to give great confidence to both surgeon and patient, that these tumors never return; once removed they give no further trouble.

M. Nelatin, therefore, excised the altered part of the maxilla. An incision of about five centimetres long was made on the level and in the direction of the base of the line. The soft parts were then held aside by the aid of a spatula, and then, with a chain saw introduced by this opening into the buccal cavity, the portion of bone invaded by the tumor was removed. This was enclosed in a bony shell, a fact which shows that it is not always possible to get the characteristic crepitation, for, as we have said, this symptom was altogether absent. The surface of a section of this cyst was free from being uniform; the central portion seemed to be made up of unchanged bony tissue, enveloped with a red matter resembling the tissue of the kidney or the spleen, and, in some points, of a whitish appearplastic elements, of medulla cells, of vessels and fibres of cellular tissue and sometimes of amorphous matter.

The epùlides, called erectile or embossed, bluish, attacking the maxilla, and often the epluides called cancerous, have for their essential element mycloplaxes, a normal element of bones, (which is hypertrophied under these circumstances and is multiplied,) and also of fibroplastic elements, medullary cells, fibres of cellular tissue and numerous vessels. Having for its fundamental element one which exists normally in bone, it is not surprising that the removed tumor returns. It is this recurrence and the physical characters of the tumor which have caused it to be confounded with cancer. As a general thing, they do not return when the operation has left no diseased tissue in the bone. It is easy to understand that, the point of departure of the disease being an element of the medulla of the bones, these should be invaded by the tumor; but what is especially to be noted is the absorption of the bony tissue before the soft tissue as it increases.

ance, seeming to be composed of an intricate interlacement of bony spiculæ. The operation was successful. On the 12th of February the girl left the Hospital completely cured. In this rapid description, we have purposely neglected some remarkable particulars, intending to return at the close of the second case, to the facts worthy of special notice.

Case II.—B., a gardener, 27 years of age, entered the hospital on the 7th of last January, having a considerable tumor on his lower jaw. His constitution is strong, and he has never had a serious illness. Thirteen months ago, for the first time, he felt a pretty acute pain in the part which corresponds to the socket of the left canine tooth. Some time afterwards, a tumor of the size of a nut appeared at this point. The physician who treated it, diagnosticated a serous cyst of the maxilla, and punctured it. A clear, limpid liquid, so the patient says, escaped, the pouch closed and resumed its growth; another puncture, with the injection of iodine, reappearance of the tumor; the same operation, the same failure. Then the physician decided to open freely the cyst, and to cauterize the inner wall with nitrate of silver. In spite of all, the tumor assumed so great a development that the patient determined to come to Paris. He was admitted in the Hospital des Cliniques.

On his entrance, we found a voluminous tumor occupying the left side of the jaw for a considerable distance. The projection on the external aspect of the lower jaw, begins on a level with the second molar, forming behind a well marked elevation, which insensibly diminishes up to the canine of the right side; that is, more than half the body of the maxilla is occupied by the tumor. Inside the projection is less; it begins near the first molar of the left side and stops at the median line of the bone. This tumor, enveloped by a bony shell which is not everywhere continuous, communicates to the finger an obscure sensation of fluctuation. This symptom, together with the information given by the patient, and the physician who attended him, seemed to point out a serous cyst. Nevertheless, the aspect of the tumor, its progress, the resistance it offered to treatment, the very doubtful sensation of fluctuation, induced M. Nelatin to believe that there might be some error in the original diagnosis. In making out his plan of treatment, he stated that he was quite prepared to find there a tumor similar to that which he had taken away a few days before.

A long incision was made upon the external face of the tumor, so as to lay bare the inner wall of the cyst. Then it was easy to see how proper was M. Nelatin's doubt as to the existence of a serous cyst. The bony shell being opened, a tumor, analogous to that last described, was found. The body of the maxilla not being completely invaded, and still being able to oppose considerable resistance, all the sound part was respected, the rest was removed by the aid of a strong gouge. The operation was completed by the application of a caustic plaster of chloride of zinc, a caustic rendered easy of application since M. Sommé, *interne en pharmacie* of the Parisian hospitals, conceived the happy idea of making a paste of it by combining it with gluten. The chloride of zinc, allowed to remain for three hours, destroyed all the parts invaded by the degeneration, which the cutting instrument could not reach.

The removed tumor had an appearance and composition precisely similar to the appearance and structure of the tumor previously described.

The consequences of the operation were fortunate; the general health satisfactory and no local accidents. The eschars occasioned by the caustic, have separated; a bony plate, very strong, especially at the base, remains and presents immense advantages, of which we shall presently speak. Everything inspires the hope that this patient will soon leave the hospital, perfectly well.

We now pass on to some important considerations.

The young girl who forms the subject of the first case, presents a deformity predicted by M. Nelatin; a deformity impossible to avoid, but nevertheless susceptible of improvement. M. A. Preterre is engaged upon it. A part of the body of the lower jaw having been cut away, the left ascending ramus and the remains of the body, deprived of fixed points, and floating, as it were, in the interior of the buccal cavity, have obeyed the muscular fibres exerted upon them, and followed the direction thus impressed. The ascending ramus is drawn a little inwards ; the right portion, more considerably, has undergone a motion of rotation and has been carried to the left, backwards and inwards, so that of the teeth implanted in this jaw, only the first and second molars and the second bicuspid correspond with those of the upper jaw, and even this contact is incomplete. The second bicuspid resting upon the right upper canine, the rest of the lower dental arch comes immediately within the upper when the jaws are closed. Mastication is, therefore, gravely compromised, and as we said before, it was impossible to avoid this deviation. M. Nelatin has shown that for this purpose a fixed point is indispensable. Now where shall we find this fixed point, both sections of the jaw hanging loose? Let us remember, also, that the chain-saw has influenced the reunion of these two parts, that is to say, that the ramus, covered with soft parts, and movable at both ends, could be of no use.

The soft parts have followed the direction of the bone, the mouth has deviated to the left, the cheek of this side is more salient, an irremediable deformity, as may be readily understood, and the more apparent, since the cheek of the opposite side is flattened ; the sole resource, therefore, is to re-establish mastication. M. Preterre has long since solved this problem, all that remains is to apply it to the present case, and that he proposes to do.

In the second case, the patient having preserved a portion of jaw entire, and strong enough to furnish a solid fixed point, he is not exposed to the danger of a similar deformity. Besides, this plate is strong enough in front to furnish a surface of implantation to the canine of the right side, and to the first incisor of the left. The second incisor and the second bicuspid of this side have been preserved and still remain, but the roots are all almost wholly uncovered; so it is probable he will lose them, as has already been the case with the canine and the other molars. It will suffice, therefore, to put in a piece carrying these teeth, to remedy all defects; still it is not indispensable, the right side being able alone to carry on the function of mastication. From these facts, we can draw the practical lesson, to assure ourselves before every operation, of the extent of the alteration of the bone, so as to preserve, if possible, a portion of the jaw, as in the second patient named above; and to cut away no more than is absolutely necessary.

As for diagnosis, we may lay down this rule, to which M. Nelatin called attention in his remarks upon the case just alluded to: always to attach more importance to the signs which we observe ourselves, than to those furnished us by patients, or even by the physicians who have attended them.

